# Infected Walled-Off Necrosis Following COVID-19-Associated Acute Pancreatitis

**DOI:** 10.7759/cureus.51889

**Published:** 2024-01-08

**Authors:** Shota Yagi, Hideki Miwa, Yosuke Kobayashi, Kenta Mitsusada

**Affiliations:** 1 Department of Respiratory Medicine, Seirei Hamamatsu General Hospital, Hamamatsu, JPN; 2 Department of Respirology, Graduate School of Medicine, Chiba University, Chiba, JPN; 3 Department of Gastroenterology, Seirei Hamamatsu General Hospital, Hamamatsu, JPN; 4 Department of Emergency Medicine, Seirei Hamamatsu General Hospital, Hamamatsu, JPN

**Keywords:** walled-off necrosis, sars-cov-2, idiopathic pancreatitis, covid-19, acute pancreatitis

## Abstract

A 54-year-old man was admitted for fever and dyspnea. He presented with severe COVID-19 pneumonia and elevated amylase and lipase levels. He received treatment for COVID-19 and possible acute pancreatitis (AP). Although pneumonia and amylase levels improved, a high-grade fever persisted. On day 39, abdominal CT revealed heterogenous liquid and non-liquid components with a well-defined wall around the pancreas, and he was diagnosed with infected walled-off necrosis (WON) after AP. It was concluded to be associated with COVID-19 because there were no identifiable causes, such as alcohol consumption, gallstones, or other viral infections. The necrotic collection and fever improved after endoscopic transgastric drainage and necrosectomy. SARS-CoV-2 is becoming recognized as a new etiological infectious factor for AP, and COVID-19-associated AP shows higher severity and mortality. Clinicians should evaluate COVID-19 patients for concomitant AP, and if it is present, they should carefully monitor the development of local complications, including WON.

## Introduction

SARS-CoV-2 has caused the ongoing COVID-19 pandemic, resulting in an enormous healthcare burden worldwide. Coronaviruses are a large family of single-stranded RNA viruses that infect humans and animals, causing a myriad of symptoms [1].

Acute pancreatitis (AP) is a common disease often caused by gallstones and alcohol abuse and is rarely of viral origin. Common causes of viral pancreatitis are cytomegalovirus, mumps, Epstein-Barr virus, Coxsackie B virus, and hepatitis A virus [2]. SARS-CoV-2 is also a causative agent of viral pancreatitis. While the complication rates of AP in patients with COVID-19 have been reported to be 0.007-0.27% [1,3], a far greater proportion of patients with COVID-19 have no identifiable cause of pancreatitis than those without COVID-19, suggesting that SARS-CoV-2 plays a causative role [3,4].

AP is morphologically classified as interstitial edematous pancreatitis, characterized by interstitial edema and necrotizing pancreatitis accompanied by extensive fat and parenchymal necrosis inside and outside the pancreas, accounting for 5-10% of patients developing necrotizing pancreatitis [5]. Walled-off necrosis (WON) is a term used to describe encapsulated collections of fluid and solid debris [6]. WON usually occurs >4 weeks after the onset of necrotizing pancreatitis and can cause pain and mechanical compression of adjacent organs and structures, leading to infection, sepsis, and multiorgan failure [5]. Although some cases of necrotizing pancreatitis have been associated with COVID-19, there have been no reports of WON after AP in our search. This is the first case report of WON following AP associated with COVID-19.

## Case presentation

**Figure 1:**
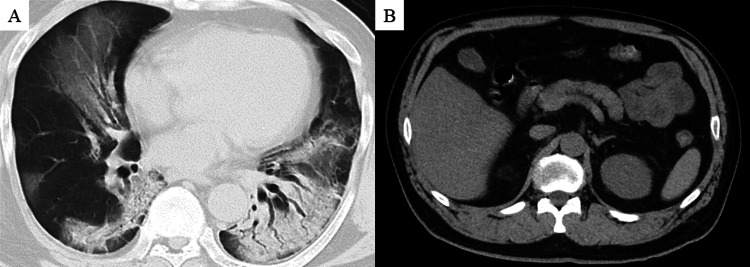
Chest and abdominal CT scans on admission (A) Chest CT scan shows bilateral ground-glass opacities and consolidation in both lungs. (B) Abdominal CT scan reveals normal gall bladder and biliary tract, with unremarkable pancreas CT: computed tomography

A 54-year-old obese patient (body mass index, 31.1 kg/m^2^) with a five-day history of fever and dyspnea was admitted to our hospital. He had a medical history of hypertension, hyperuricemia, and bronchial asthma. He reported no history of alcohol consumption or smoking. Upon admission, the physical examination results, including chest auscultation and epigastric pain, were unremarkable. His body temperature was 38.3℃, blood pressure 127/100 mmHg, and oxygen saturation 77% on room air. The patient tested positive for SARS-CoV-2 RNA. Laboratory tests upon admission revealed elevated levels of aspartate aminotransferase (154 U/L), alanine aminotransferase (94 U/L), amylase (1,083 U/L), lipase (543 U/L), creatinine (1.27 mg/dL), and C-reactive protein (17.7 mg/dL). Plasma triglyceride and calcium levels were normal. Computed tomography (CT) revealed bilateral ground-glass opacities and consolidation in both lungs (Figure 1A) and a normal gall bladder and biliary tract, with an unremarkable pancreas (Figure 1B).

**Figure 2:**
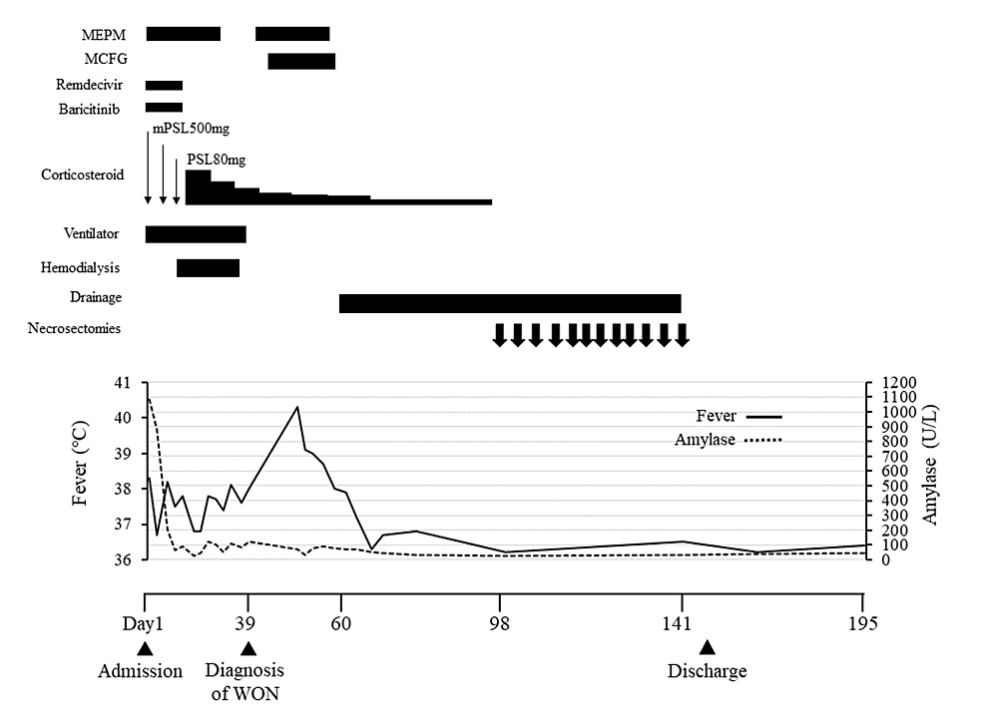
Clinical course after admission Although the respiratory and renal impairments gradually improved, high-grade fever persisted until treatment for WON was initiated MCFG: micafungin; MEPM: meropenem; mPSL: methylprednisolone; PSL: prednisolone; WON: walled-off necrosis

The patient was intubated and admitted to the intensive care unit for mechanical ventilation. He was administered high-dose corticosteroids, remdesivir, baricitinib, and unfractionated heparin. He also received aggressive fluid administration and empiric antibiotics for the possibility of AP and bacterial pneumonia. Nutritional support was initiated through a nasojejunal tube. On day 6, an abdominal ultrasound revealed fluid accumulation around the pancreas, supporting the diagnosis of AP. Gallstones were not observed. Additionally, the patient developed acute kidney failure and required hemodialysis (Figure 2). In contrast, circulatory dynamics were preserved without catecholamines.

**Figure 3:**
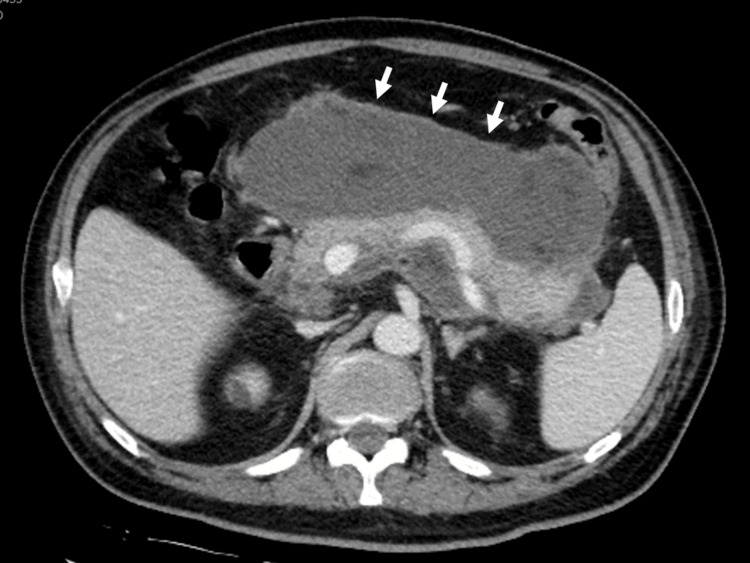
Abdominal CT scans at diagnosis of WON CT scans of the abdomen show a heterogenous collection showing liquid and non-liquid components with a well-defined wall around the pancreas (white arrow) CT: computed tomography, WON: walled-off necrosis

**Figure 4:**
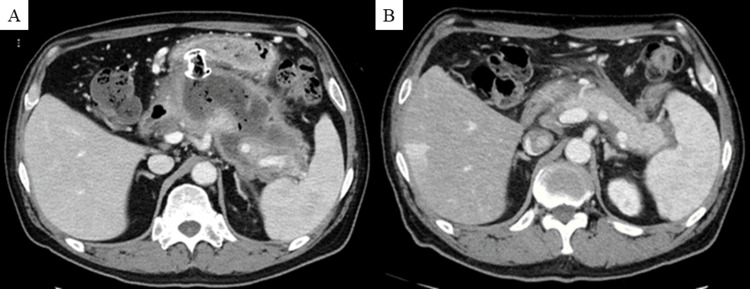
Changes in abdominal CT scans after initiation of treatment for WON (A) Endoscopic ultrasound-guided transgastric drainage using a lumen-apposing metal stent did not improve encapsulated collection significantly (day 98). (B) A follow-up CT after endoscopic necrosectomy shows marked improvement in the encapsulation collection (day 195) CT: computed tomography, WON: walled-off necrosis

Thereafter, the respiratory and renal impairments gradually improved. Amylase levels also decreased after admission. The patient was weaned from hemodialysis on day 32 and received ventilation on day 39 (Figure 2). However, the high-grade fever persisted, and abdominal CT demonstrated a heterogenous collection showing liquid and non-liquid components with a well-defined wall around the pancreas (Figure 3), leading to the diagnosis of WON. Although the radiological findings were unremarkable upon admission, he was assumed to have necrotizing pancreatitis. No other causes of AP, including alcohol consumption, gallstones, other viral infections, hypertriglyceridemia, or hypercalcemia, were identified. Antifungal therapy for candidemia due to catheter-related bloodstream infections did not improve the fever. Therefore, the patient was diagnosed with infected WON following COVID-19-associated AP. On day 60, the patient underwent endoscopic ultrasound-guided transgastric drainage using a lumen-apposing metal stent, which improved his fever. However, the encapsulated collection did not improve significantly (Figure 4A). Therefore, a direct endoscopic necrosectomy was also performed (12 sessions).

After endoscopic necrosectomy, the encapsulated collection was markedly improved. Bilateral ground-glass opacities and consolidation in the lung field were also improved, and oxygen therapy was no longer necessary. On day 150, the patient was discharged to his home. A follow-up CT showed that the encapsulation collection remained improved (Figure 4B). The patient had no symptoms at six months of follow-up.

## Discussion

Upon admission, the patient presented with severe COVID-19 pneumonia, acute kidney failure, and elevated amylase and lipase levels. Although the abdominal CT did not show supportive findings for AP, the patient underwent aggressive fluid resuscitation and nutritional support through a nasojejunal tube for possible AP and COVID-19 treatment. Although pneumonia and amylase levels improved, a high-grade fever persisted, and the patient was diagnosed with infected WON. The absence of other etiologies suggests that AP is associated with COVID-19. Our report is the first case of WON following AP associated with COVID-19. Researchers believe that this case provides important information regarding the comorbidities associated with COVID-19.

The most common causes of AP are gallstones and excessive alcohol consumption. Hypertriglyceridemia, endoscopic retrograde cholangiopancreatography (ERCP), drugs, and viral infections (e.g., cytomegalovirus, mumps, Epstein-Barr virus, Coxsackie B virus, and hepatitis A virus) can also cause AP [2]. In contrast, idiopathic pancreatitis, the etiology of which is unknown, is reported in 10-30% of all AP cases [7]. Some reports revealed a high incidence of idiopathic pancreatitis in patients with COVID-19, suggesting a relationship between COVID-19 and AP. Inamdar et al. showed that idiopathic pancreatitis was the most common etiology (68.8%) in patients with AP who tested positive for COVID-19, compared with 21.0% in patients who were COVID-19 negative [3]. de-Madaria et al. also reported that patients with COVID-19 had a significantly higher rate of idiopathic pancreatitis than those without (24% vs. 14%, p=0.001) [4]. Silva et al. suggested that SARS-CoV-2 infection may be responsible for AP in certain patients, provided other known etiologies are ruled out [8]. Our patient had no history of alcohol consumption, gallstones, hypertriglyceridemia, ERCP, or drug use prior to admission. Other viral infections (e.g., cytomegalovirus, Epstein-Barr virus) were excluded based on blood tests and medical history. The current case corresponds to idiopathic pancreatitis, which suggests an association between COVID-19 and AP.

Although the association between the severity of COVID-19 pneumonia and the development of AP remains unknown, high rates of severe AP are observed in patients with COVID-19. A meta-analysis showed that COVID-19-associated AP has a higher incidence of necrotizing pancreatitis, higher severity, and higher mortality than observed among patients with non-COVID-19 AP [9]. Clinicians should check amylase levels on blood tests for the following COVID-19 patients: first, in patients with epigastric pain; and second, when abdominal symptoms and findings are difficult to confirm due to emergent medical care (including mechanical ventilation). They should also pay attention to the severity of the AP.

Direct pancreatic injury via angiotensin-converting enzyme-2 (ACE2) has been suggested as the mechanism underlying AP in patients with COVID-19. The attachment of the virus to ACE2 in lung type 2 alveolar cells induces lung injury [10]. ACE2 expression is slightly higher in the pancreas than in the lungs, possibly associated with pancreatic damage following the SARS-CoV-2 infection [11]. SARS-CoV-2 has been found in necrotic abdominal and pancreatic pseudocyst (PPC) fluid samples from patients with SARS-CoV2-associated pneumonia and AP [12]. Our patient had renal failure in addition to pancreatic damage. In the kidney, ACE2 is abundantly expressed in podocytes and proximal straight tubule cells [13]. SARS-CoV-2 was confirmed in the tubular epithelium and podocytes by the autopsy of a patient who died of COVID-19 [14]. In addition, the SARS-CoV-2 S protein binds to human ACE2 with a higher affinity than the SARS-CoV (coronavirus that initially emerged in China and caused the 2002-04 SARS outbreak) S protein [15]. As mentioned above, direct ACE2-mediated impairment has been suggested as a mechanism of pancreatic and renal injury in patients with COVID-19, and a similar mechanism was likely to occur in this case. Another mechanism of pathogenesis may be multiorgan failure due to a COVID-19 infection-induced cytokine storm [8]. However, our patient did not have elevated blood lactate levels, hypotension, or disseminated intravascular coagulation (DIC).

Pancreatic fluid collection (PFC) is a common local complication in AP. The revised Atlanta classification categorizes PFCs into acute peripancreatic fluid collection, acute necrotic collection, PPC, and WON depending on the time after the onset of AP (≤4 vs. >4 weeks) and the presence of necrosis [16]. PPC contains homogenous fluid collections, and WON is a heterogenous encapsulated collection with fluid and non-liquid components [6]. In this case, we diagnosed WON because it showed an encapsulated heterogenous fluid collection containing necrotic materials. WON can cause pain and mechanical compression of adjacent organs and structures, leading to infection, sepsis, and multiorgan failure [5]. Heckler et al. revealed that there are two phases of mortality, and although the primary cause of death in the first week is multiorgan failure, most subsequent deaths are due to local pancreatic necrosis [17]. Therefore, the management of local complications of AP is crucial.

Although the risk factors for WON in patients with necrotizing pancreatitis are not clearly known, Ikarashi et al. revealed that high body mass index (≥25 kg/m^2^), post-ERCP pancreatitis, and DIC are risk factors for the development of WON associated with severe AP [18]. Our patient was severely obese but did not develop DIC and had no history of ERCP. In obese patients, elevated levels of proinflammatory mediators, decreased levels of anti-inflammatory cytokines, and large amounts of necrotic abdominal fat may lead to local complications [18]. In the present case, obesity may have been a factor in the development of the WON. Although a multicenter cohort study showed that hypertriglyceridemia is an independent risk factor for acute necrotic collection and WON [19], and a systematic review and meta-analysis also showed a higher incidence of pancreatic necrosis in the hypertriglyceridemia group [20], triglyceride levels were normal in our patients.

## Conclusions

We report the first case of infected WON following COVID-19-associated AP. Patients with COVID-19 have a higher rate of idiopathic pancreatitis, in which other known factors have been ruled out than observed among non-COVID-19 patients. Therefore, SARS-CoV-2 is being recognized as a new etiological infectious factor for AP. Moreover, COVID-19-associated AP shows higher severity and mortality. Clinicians should evaluate COVID-19 patients for concomitant AP, and if it is present, they should carefully monitor the development of local complications, including WON.

## References

[1] Miró Ò, Llorens P, Jiménez S (2020). Frequency of five unusual presentations in patients with COVID-19: results of the UMC-19-S(1). Epidemiol Infect.

[2] Forsmark CE, Vege SS, Wilcox CM (2016). Acute pancreatitis. N Engl J Med.

[3] Inamdar S, Benias PC, Liu Y, Sejpal DV, Satapathy SK, Trindade AJ (2020). Prevalence, risk factors, and outcomes of hospitalized patients with coronavirus disease 2019 presenting as acute pancreatitis. Gastroenterology.

[4] de-Madaria E, Capurso G (2021). COVID-19 and acute pancreatitis: examining the causality. Nat Rev Gastroenterol Hepatol.

[5] Banks PA, Bollen TL, Dervenis C (2013). Classification of acute pancreatitis--2012: revision of the Atlanta classification and definitions by international consensus. Gut.

[6] Gardner TB, Coelho-Prabhu N, Gordon SR (2011). Direct endoscopic necrosectomy for the treatment of walled-off pancreatic necrosis: results from a multicenter U.S. series. Gastrointest Endosc.

[7] Mazza S, Elvo B, Conti CB (2022). Endoscopic ultrasound diagnostic gain over computed tomography and magnetic resonance cholangiopancreatography in defining etiology of idiopathic acute pancreatitis. World J Gastrointest Endosc.

[8] Silva JT, Fonseca Neto OC (2023). Acute pancreatitis and COVID-19: an integrative review of the literature. Rev Col Bras Cir.

[9] Aziz AA, Aziz MA, Omar N, Saleem M, Pahuja KH, Haseeb Ul Rasool M, Shah R (2023). A meta-analysis of the severity of acute pancreatitis (AP) in COVID-19 infection. Cureus.

[10] Beyerstedt S, Casaro EB, Rangel ÉB (2021). COVID-19: angiotensin-converting enzyme 2 (ACE2) expression and tissue susceptibility to SARS-CoV-2 infection. Eur J Clin Microbiol Infect Dis.

[11] Liu F, Long X, Zhang B, Zhang W, Chen X, Zhang Z (2020). ACE2 expression in pancreas may cause pancreatic damage after SARS-CoV-2 infection. Clin Gastroenterol Hepatol.

[12] Aday U, Gedik E, Kafadar MT, Özbek E (2021). Acute necrotizing pancreatitis and coronavirus disease-2019 (COVID-19). Korean J Gastroenterol.

[13] Pan XW, Xu D, Zhang H, Zhou W, Wang LH, Cui XG (2020). Identification of a potential mechanism of acute kidney injury during the COVID-19 outbreak: a study based on single-cell transcriptome analysis. Intensive Care Med.

[14] Su H, Yang M, Wan C (2020). Renal histopathological analysis of 26 postmortem findings of patients with COVID-19 in China. Kidney Int.

[15] Wang Q, Zhang Y, Wu L (2020). Structural and functional basis of SARS-CoV-2 entry by using human ACE2. Cell.

[16] Nakai Y, Shiomi H, Hamada T (2023). Early versus delayed interventions for necrotizing pancreatitis: a systematic review and meta-analysis. DEN Open.

[17] Heckler M, Hackert T, Hu K, Halloran CM, Büchler MW, Neoptolemos JP (2021). Severe acute pancreatitis: surgical indications and treatment. Langenbecks Arch Surg.

[18] Ikarashi S, Kawai H, Hayashi K (2020). Risk factors for walled-off necrosis associated with severe acute pancreatitis: a multicenter retrospective observational study. J Hepatobiliary Pancreat Sci.

[19] Song K, Wu Z, Meng J (2023). Hypertriglyceridemia as a risk factor for complications of acute pancreatitis and the development of a severity prediction model. HPB (Oxford).

[20] Kiss L, Fűr G, Mátrai P (2018). The effect of serum triglyceride concentration on the outcome of acute pancreatitis: systematic review and meta-analysis. Sci Rep.

